# Effects of Fe, Mn Individual Doping and (Fe, Mn) Co-Doping on Ferromagnetic Properties of Co_2_Si Powders

**DOI:** 10.3390/nano12020293

**Published:** 2022-01-17

**Authors:** Jiang Zou, Lifeng Wang, Juan He, Bo Wu, Quan Xie

**Affiliations:** 1 College of Big Data and Information Engineering, Guizhou University, Guiyang 550025, China; yywanj1@163.com; 2 School of Physics and Electronic Science, Zunyi Normal College, Zunyi 563006, China; wlf1234560116@163.com (L.W.); hejuan0209@126.com (J.H.); fqwubo@163.com (B.W.)

**Keywords:** magnetic, doping, Co_2_Si, saturation magnetisation, coercivity, remanence

## Abstract

Magnetic materials are crucial energy materials that are widely used in day-to-day life. Therefore, the development and study of high-performance magnetic materials are of great significance. In this study, the magnetic materials Co${}_{66.6}$Si${}_{33.4}$, Co${}_{60.6}$X${}_{6}$Si${}_{33.4}$ (X = Fe, Mn), and Co${}_{60.6}$Fe${}_{3}$Mn${}_{3}$Si${}_{33.4}$ were prepared via the ball milling and sintering processes. Their crystal structures, electrical conductivity, and magnetic properties were investigated via the X-ray diffraction analysis and by using a resistivity tester, vibrating sample magnetometer, and vector network analyser. The X-ray diffraction analysis revealed that a single phase of Co${}_{66.6}$Si${}_{33.4}$ and its doped alloy powders were successfully obtained. The electrical conductivities of Mn${}_{6}$Co${}_{60.6}$Si${}_{33.4}$ and Fe${}_{3}$Mn${}_{3}$Co${}_{60.6}$Si${}_{33.4}$ were measured using a resistivity tester. The results indicate that Mn doping and Fe and Mn Co-doping enhanced the electrical conductivity of Co${}_{66.6}$Si${}_{33.4}$. The magnetic properties of Co${}_{66.6}$Si${}_{33.4}$ were determined using a vibrating sample magnetometer. We observed that the magnetic properties were enhanced after doping. Co${}_{60.6}$Fe${}_{3}$Mn${}_{3}$Si${}_{33.4}$ exhibited excellent magnetic properties. Further, its permeability was determined using a vector network analyser. At a low frequency, the u’ and u” values of Co${}_{60.6}$Fe${}_{6}$Si${}_{33.4}$ and Co${}_{60.6}$Fe${}_{3}$Mn${}_{3}$Si${}_{33.4}$ were enhanced; whereas, at a high frequency, after doping, the u’ and u” values changed only slightly. This study can be used as a basis for future studies on magnetic functional materials.

## 1. Introduction

Magnetic materials are widely used in various fields of renewable energy, energy conservation and environmental protection, such as solar and wind power generation, new energy vehicles, rail transit, energy saving lighting, and new planar displays [1,2,3]. Presently, the intermetallic compound Co-Si is an important alloy widely used in the fields of electronics, power, and machinery [4,5]. It has good oxidation and corrosion resistances, stable physical and chemical properties, high temperature resistance, high strength, a large elastic modulus, and high structural stability [6]. It has the potential to be used as a high-temperature structural material.

Chuan et al. [7] prepared CoSi by mechanical alloying and performed thermodynamic analysis. They observed that metallic compounds were initially formed. PanzhiJun et al. [8] studied the thermoelectric properties of CoSi. Joa et al. investigated the magnetic properties of Co–Si alloy clusters. The electronic structure and magnetic properties of the Co–Si alloy clusters were investigated using ab initio spin-polarised density functional calculations. The magnetic moment primarily depended on the Co–Si bond lengths rather than the Co–Co bond lengths [9].

Velez and Valvidares conducted a study on the structural and magnetic properties of amorphous Co–Si alloy films. The results showed that the heterogeneity of the high Co content led to an essentially isotropic magnetic behaviour and the formation of highly coercive fields [10].

Geller synthesized single-phase Co${}_{2}$Si by mechanical ball milling and heat treatment and determined its crystal structure and magnetic properties [11]. China-hong investigated the following three intermediate phases: Co${}_{2}$Si, CoSi, and CoSi${}_{2}$. The results showed that CoSi, Co${}_{2}$Si, and CoSi${}_{2}$ were found in these couples when the bulks were annealed at temperatures of 800, 900, 1000, and 1050 °C [12]. Cristina Bormio-Nunes studied the magnetisation of the Co–Si-B system. The results of the study showed that Co${}_{2}$Si is paramagnetic [13].

Tu et al. investigated the thermal stability and growth kinetics of Co${}_{2}$Si and CoSi in thin-film reactions. The results showed that a sequential growth of Co${}_{2}$Si and CoSi was observed in the reaction between the Si and Co films. First, Co${}_{2}$Si was formed, and, subsequently, CoSi was formed when all of the Co was consumed [14]. Baldan et al. investigated the microstructural evidence of β Co${}_{2}$Si-phase stability in the Co–Si System. The stability of the β Co${}_{2}$Si phase in the Co–Si system was verified [15].

Vander Walls-Zeeman studied the atomically disordered nanocrystalline compound Co${}_{2}$Si prepared via high-energy ball milling. The results indicated anti-site disorder. Moreover, the continuous increase in the rate of magnetisation confirmed the generation of the anti-site disorder in Co${}_{2}$Si [16].

However, studies on enhancing the magnetic properties of Co${}_{2}$Si by doping have not been reported thus far. In this study, Co${}_{2}$Si was prepared by ball milling with Fe and Mn as dopants and, Fe and Mn as co-dopants. The improvements in its magnetic properties were investigated.

## 2. Experimental Details

Co${}_{66.6}$Si${}_{33.4}$, Co${}_{60.6}$X${}_{6}$Si${}_{33.4}$ (X = Fe, Mn), and Co${}_{60.6}$Fe${}_{3}$Mn${}_{3}$Si${}_{33.4}$ were prepared using Co, Si, Fe, and Mn powders with purity of 99.9% according to their molar fractions [17]. This method can only be used for inactive metals at room temperature to prevent severe oxidation in a short time. The powders after accurate weighing were placed in a stainless steel tank for ball milling. An OECO-PBM-AD-6L omni-directional ball mill manufactured by Hunan Deke was used to mill the powder. The samples were added with a ball-to-powder weight ratio of 40:1, and milling was performed for a duration of 72 h.

The rotational speed of the ball milling machine was maintained at 360 rpm. To prevent oxidation during ball milling, the stainless-steel tank was evacuated insert and pressed into a pellet [18]. Furthermore, close attention had to be paid to the sealing of the steel tank to prevent air leakage. The alloy powder obtained by ball milling was annealed at 950 °C for 2 h in a GSL-1500X tube furnace (Hefei Kejing Material Technology Co, Ltd. He Fei, Anhui, China), which incorporates a double layer air cooling structure to sinter samples.

The maximum sintering temperature of GSL-1500X tube furnace was 1500 °C. The structure of the sintered samples was characterised by X-ray diffraction (XRD, BRUKER D8 ADVANCE (Zhongguancun, Beijing, China), which is produced by the technology company BRUKER [19] to detect the phase of the material. To ensure the accuracy of the diffraction data, first, the sample was ground into a powder suitable for the diffraction experiment; subsequently, sample powder was transformed into a flat test piece.

The hysteresis loop of each samples was measured using a vibrating sample magnetometer (VSM, Lakeshore7404, Baoshan District, Shanghai, China) [20]. When the hysteresis loop of a nanomagnetic material was being tested, as the material had a considerably small particle size and rich surface, the magnetisation of the surface magnetic moment of the magnetic particle was difficult to measure due to its high energy. This led to the hysteresis loop not being sufficiently stable in a high magnetic field.

Generally, the magnetisation corresponding to the maximum magnetic field is considered the saturation magnetisation of such materials. The permeability was determined using the vector network analyser (Agilent, Chaoyang District, Beijing, China) [21]. The coaxial method is the most suitable for powders and the waveguide method is primarily suitable for resins. In the course of measurement, a coaxial ring was made of powder and paraffin. The concentration ratio of sample to paraffin was 40:60. The conductivity of the samples was measured using a high-precision resistivity tester (YAOS, Guangzhou, GuangDong, China) [22].

The powder micrograph images were tested by transmission electron microscopy (TEM; Tecnai G2 F20, Fei Company, Hillsboro, OR, USA) [23]. The TEM test showed the micrograph images and grain size on the nanoscale. The sample comprised organic or magnetic matter; therefore, the turbulence under high pressure was more severe. Consequently, it was difficult to capture a good effect, leading to errors.

## 3. Results and Discussion

**Figure 1:**
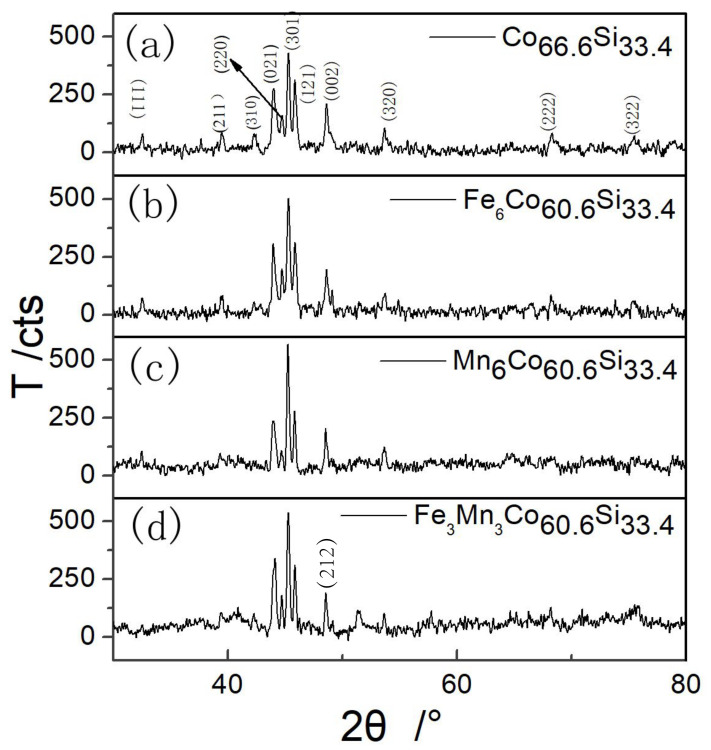
XRD patterns of (**a**) as-prepared Co${}_{66.6}$Si${}_{33.4}$, (**b**) Fe doped Co${}_{66.6}$Si${}_{33.4}$, (**c**) Mn doped Co${}_{66.6}$Si${}_{33.4}$ and (**d**) Fe and Mn co-doped Co${}_{66.6}$Si${}_{33.4}$.

Figure 1 shows the XRD pattern of mechanically alloyed powder after sintering at 950 °C. All the highly intense peaks match those of the diffraction pattern of Co${}_{2}$Si with the crystal system of orthorhombic (pdfcard98-005-2281).

As shown in Figure 1, highly intense diffraction peaks were observed corresponding to (021), (301) and (121) planes. From the XRD pattern of Co${}_{66.6}$Si${}_{33.4}$ shown in Figure 1a, no diffraction peaks of Co and Si appeared after sintering the samples at 950 °C, indicating that the synthesized powder comprised a single Co${}_{66.6}$Si${}_{33.4}$ phase after mechanical alloying and heat treatment. Figure 1b–d shows the XRD patterns of X${}_{6}$Co${}_{60.6}$Si${}_{33.4}$, (X = Fe, Mn), and Fe${}_{3}$Mn${}_{3}$Co${}_{60.6}$Si${}_{33.4}$, respectively. The phase of Co${}_{66.6}$Si${}_{33.4}$ was observed by XRD. The Fe–Mn phase was not observed in the doped samples, as shown in Figure 1b–d, indicating that doping did not form other phases except the phase of Co${}_{66.6}$Si${}_{33.4}$. The diffraction peaks of (111), (211), and (310) at 32.593°, 39.459°, and 42.303°, respectively, observed for Co${}_{66.6}$Si${}_{33.4}$, were not present for Co${}_{60.6}$Fe${}_{3}$Mn${}_{3}$Si${}_{33.4}$.

In contrast, the diffraction peaks of (212) and (122) appeared at 51.327° and 58.850° for Co${}_{60.6}$Fe${}_{3}$Mn${}_{3}$Si${}_{33.4}$. These observations indicated that the doping of Mn and Fe-Mn slightly changed the preferred orientation. Table 1 shows different values of 2θ°, the error between the measured and standard diffraction angle (B), the crystallographic inter-planar spacing (d), and the lattice constants of the corresponding crystal plane (a, b, and c). B and d were obtained by the XRD analysis.

By importing the detected data of the XRD, the lattice parameters of the crystal planes were calculated using JADE6.5 [24], which is an XRD analysis software. This software performs various functions, including XRD analysis of a given material, and analysis of its lattice parameters, FWHM (the half-width height of diffraction peak), and grain size. The diffraction peaks within the 2θ range from 41° to 51° were fitted using the Pearson-VII function [25]. The crystal sizes were obtained using the Scherrer formula and the Williamson–Hall method [26]. The calculation is as follows:

(1) $$\begin{array}{c} \beta_{hkl}=\beta_{s}-\beta_{D} \end{array}$$

(2) $$\begin{array}{c} \beta_{hkl}=\left(\frac{k\lambda }{D\cos\theta }\right)+4\varepsilon \tan\theta \end{array}$$

(3) $$\begin{array}{c} \beta_{s}=4\varepsilon \tan\theta \end{array}$$

where *D* is the grain size in nanometres, $\beta_{hkl}$ is the total broadening, λ is the wavelength of the radiation, *k* is a constant equal to 0.94, ε is the strain, $\beta_{D}$ is the peak width at half-maximum intensity, and θ is the peak position.

**Figure 2:**
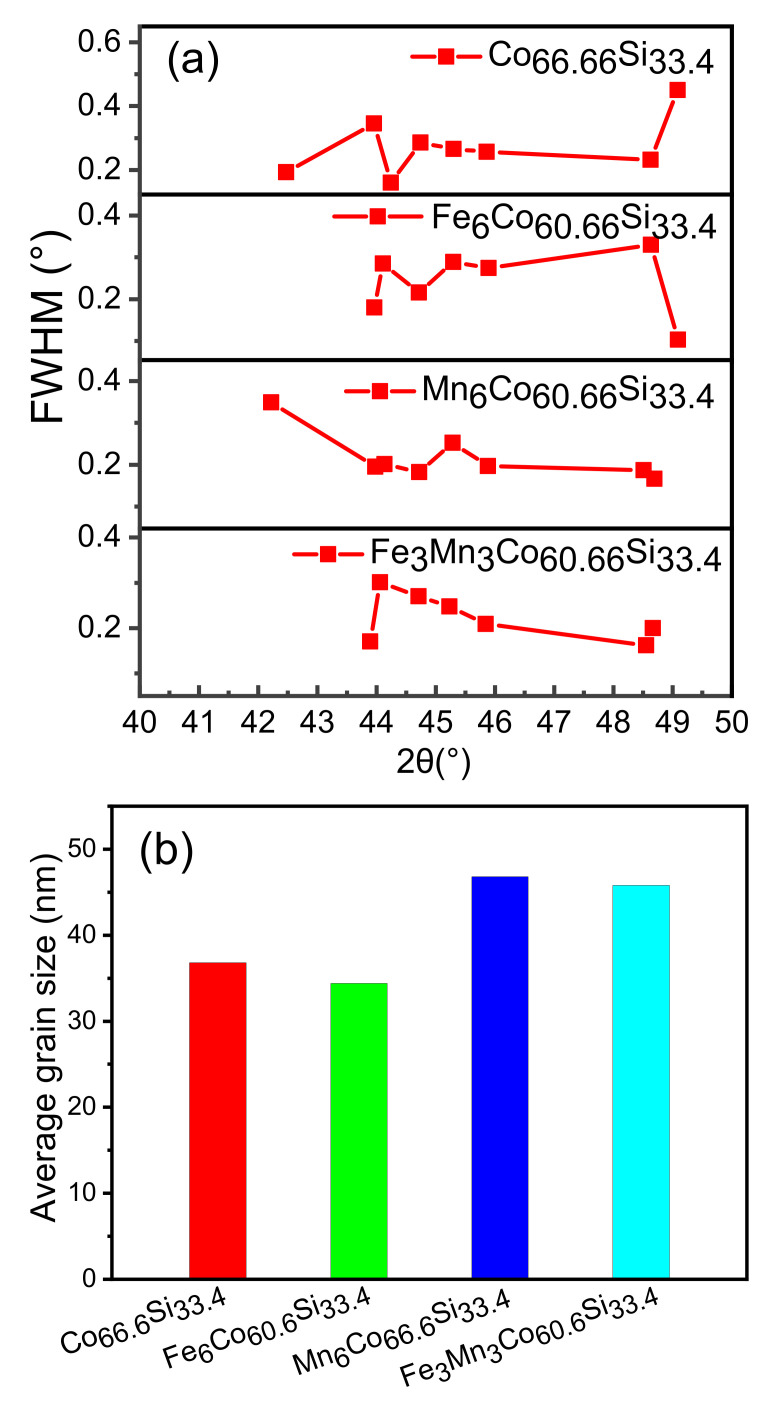
(**a**) FWHM of Co${}_{66.6}$Si${}_{33.4}$, Co${}_{60.6}$X${}_{6}$Si${}_{33.4}$, (X = Fe, Mn), and Co${}_{60.6}$Fe${}_{3}$Mn${}_{3}$Si${}_{33.4}$; (**b**) Average grain size of as-prepared Co${}_{66.6}$Si${}_{33.4}$, Co${}_{60.6}$X${}_{6}$Si${}_{33.4}$, (X = Fe, Mn), and Co${}_{60.6}$Fe${}_{3}$Mn${}_{3}$Si${}_{33.4}$.

Figure 2a shows that the FWHM of the diffraction peaks for each sample were obtained within 41–51° using JADE6.5. Figure 2b shows the average grain size calculated using the Williamson–Hall method. Samples of about 1 g were applied to the glass for XRD and smoothed. The data of the samples obtained were imported into JADE. Then the diffraction peaks, (021), (220), (301), (121), and (002), were selected to calculate its grain size due to the major contribution of grain size. The calculated average grain size was 36.8 nm for Co${}_{66.6}$Si${}_{33.4}$, and the grain size changed after doping. This indicates that doping had an influence on the growth of the grain.

According to Table 2, the measured average grain sizes were 23, 21, 38, and 32 nm, for Co${}_{66.6}$Si${}_{33.4}$, Co${}_{60.6}$X${}_{6}$Si${}_{33.4}$ (X = Fe, Mn), and Co${}_{60.6}$Fe${}_{3}$Mn${}_{3}$Si${}_{33.4}$, respectively. When the XRD was performed, the amount of powder was relatively large, about 1 g. In the XRD analysis. When TEM is made, only a small particle is taken about a few milligrams. Therefore, there was a certain error between the measured and estimated values.

**Figure 3:**
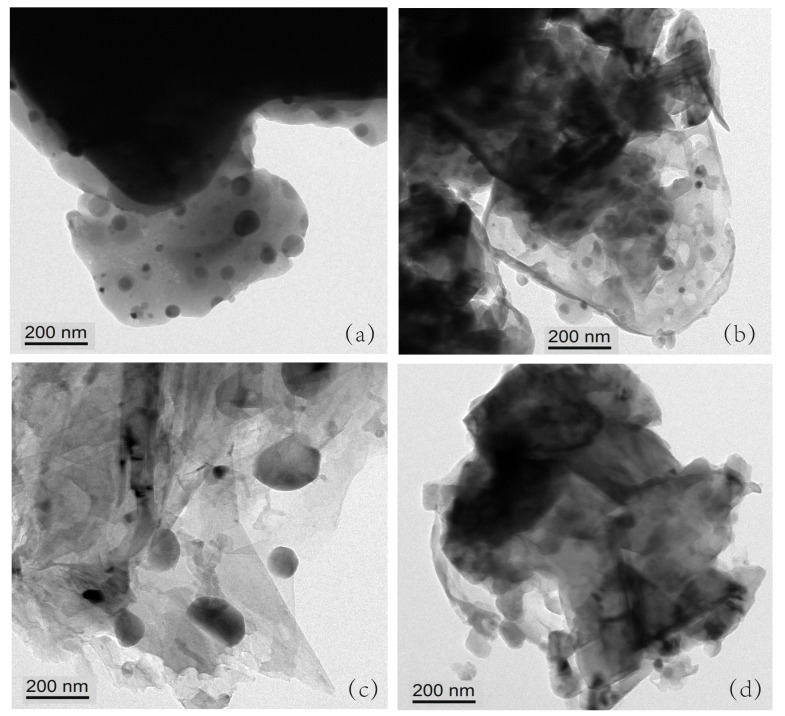
(**a**–**d**) Powder micrograph images of Co${}_{66.6}$Si${}_{33.4}$, Co${}_{60.6}$X${}_{6}$Si${}_{33.4}$, (X = Fe, Mn), and Co${}_{60.6}$Fe${}_{3}$Mn${}_{3}$Si${}_{33.4}$.

A magnetic material affects the accuracy of the testing equipment, which is one of the reasons for the error. Figure 3a–d shows the powder micrograph images of Co${}_{66.6}$Si${}_{33.4}$, Co${}_{60.6}$X${}_{6}$Si${}_{33.4}$ (X = Fe, Mn), and Co${}_{60.6}$Fe${}_{3}$Mn${}_{3}$Si${}_{33.4}$. The scale bar in Figure 3a–d equals 200 nm. The radii of Fe and Mn atoms are not greatly different from that of Co atoms. Hence, Fe and Mn atoms can enter the Co${}_{2}$Si lattice and replace the Co atoms. In the periodic table, Fe and Co are adjacent to each other.

Therefore, when Fe atoms replace the Co atoms, they have little effect on the lattice growth compared to Mn atom doping. As the radii of Fe atoms and Co atoms are not very different, the diffusion is slow during the heating process, thereby, hindering the growth of the grain. The radius of Mn is smaller than that of Fe; therefore, it is easier to enter the Co${}_{2}$Si lattice. Therefore, Mn doping and Fe–Mn doping promote growth of the grain.

Table 3 shows the conductivities of the as-prepared Co${}_{66.6}$Si${}_{33.4}$, Co${}_{60.6}$X${}_{6}$Si${}_{33.4}$ (X = Fe, Mn), and Co${}_{60.6}$Fe${}_{3}$Mn${}_{3}$Si${}_{33.4}$. To study the effect of doping on the conductivities of the as-prepared Co${}_{66.6}$Si${}_{33.4}$ and doped Co${}_{66.6}$Si${}_{33.4}$, the resistivities of the samples were measured using a high-precision resistivity tester. Ench sample was placed inside the sample pool, and a certain pressure was applied to the sample. The current and voltage parameters were set, and the high-precision resistivity tester was used to record the data. Under different pressures, the resistivities recorded were different; thus, we selected three different pressures. A resistance meter was used to measure their resistivities under the pressures of 1, 2, and 3 MPa.

The conductivity was calculated by taking the reciprocals of resistivities. As shown in Table 3, we chose 3 MPa as an example because the compounds followed the same rules under this pressure. Under 3 MPa, the conductivity of Co${}_{66.6}$Si${}_{33.4}$ was 183 S/m, which is very low. The conductivity of Fe${}_{6}$Co${}_{60.6}$Si${}_{33.4}$ was 190 S/m, which indicates that the effect of Fe doping on its conductivity is insignificant.

The conductivities of Mn${}_{6}$Co${}_{60.6}$Si${}_{33.4}$ and Fe${}_{3}$Mn${}_{3}$Co${}_{60.6}$Si${}_{33.4}$ were 111,000 and 32,000 S/m, indicating that Mn doping and Fe, Mn co-doping enhance the conductivity of Co${}_{66.6}$Si${}_{33.4}$. Conductivity is mainly determined by electron mobility. After sintering to form Co${}_{2}$Si, the Co–Si bonding with electrons becomes relatively strong, resulting in a low conductivity.

After doping, the outermost layer of the 4d orbital of the Fe atom contains six electrons, whereas that of the 4d orbital of the Mn atom contains five electrons. Further, it is easier to lose a single electron from the outermost layer of the 4d orbital than to lose two electrons. Therefore, the influence of Mn doping on improving the conductivity is significant, while that of Fe doping is relatively small.

**Figure 4:**
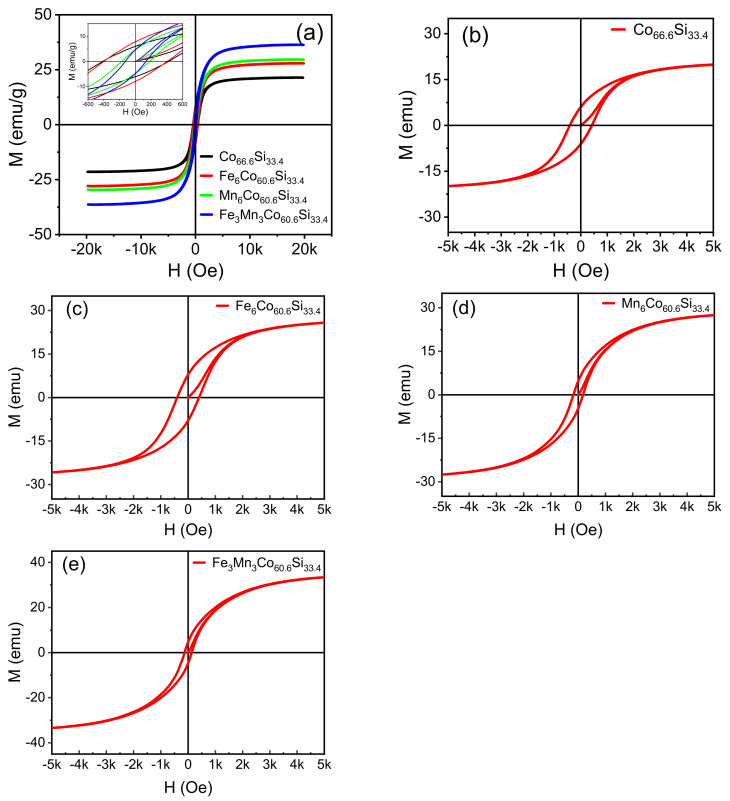
(**a**–**e**) Hysteresis loops of the as-prepared Co${}_{66.6}$Si${}_{33.4}$, Co${}_{60.6}$X${}_{6}$Si${}_{33.4}$, (X = Fe, Mn), Co${}_{60.6}$Fe${}_{3}$Mn${}_{3}$Si${}_{33.4}$ samples sintered at 950 °C.

Figure 4a–e shows the hysteresis loops of Co${}_{66.6}$Si${}_{33.4}$, Co${}_{60.6}$X${}_{6}$Si${}_{33.4}$, (X = Fe, Mn), and Co${}_{60.6}$Fe${}_{3}$Mn${}_{3}$Si${}_{33.4}$, which were observed under a parallel magnetic field. All samples exhibit magnetic properties. Cristina Bormio-Nunes [13] studied the magnetisation of Co${}_{2}$Si, which was obtained by heat treatment.

They obtained a saturation magnetisation of 18 KA/M, indicating that the magnetic properties are better than those obtained in our study. However, they performed they found powder heating at 1000 °C for 50 h, which requires complex instrumentation as Co${}_{2}$Si is easily oxidised at high temperatures. Van der Walls-Zeeman [16] obtained Co${}_{2}$Si by ball milling and sintering. They milled Co${}_{2}$Si for 120, 260, and 629 h, then sintered it at 900 K. The diffraction peak matched the diffraction pattern of Co${}_{2}$Si for the sample ball-milled for 629 h. However, the machine was continuously operated for hundreds of hours, resulting in damage to the mechanical bearing. Therefore, our method is more effective.

Magnetisation gradually increases as increase in the magnetic field intensity (H); at a certain value, it attains saturation. The doping of Co${}_{66.6}$Si${}_{33.4}$ with Fe and Mn improved the saturation magnetisation of the sample. The obtained saturation magnetisations were 27.9, 29.6, and 36.3 emu/g. The magnetic moment of Co${}_{2}$Si was significantly improved when Co${}_{66.6}$Si${}_{33.4}$ was doped with Fe and Mn, as inferred from theoretical analysis. By introducing different metal ions as dopants, the magnetism of Co${}_{66.6}$Si${}_{33.4}$ could be ameliorated.

**Figure 5:**
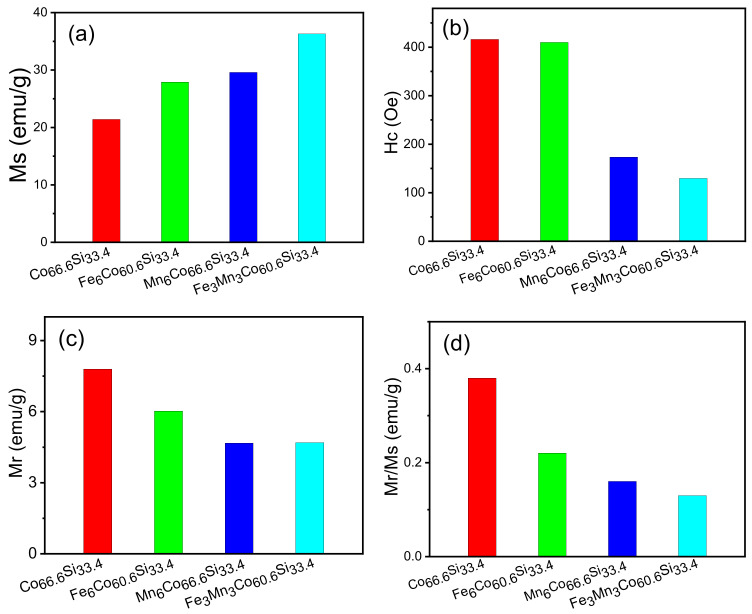
(**a**–**d**) M${}_{s}$, H${}_{c}$, M${}_{r}$ and M${}_{r}$/M${}_{s}$ of as-prepared Co${}_{66.6}$Si${}_{33.4}$, Co${}_{60.6}$X${}_{6}$Si${}_{33.4}$, (X = Fe, Mn), Co${}_{60.6}$Fe${}_{3}$Mn${}_{3}$Si${}_{33.4}$ samples.

The lattice will be changed during the introduction of ions, and in this process, some magnetic moments may be occur [27]. Consequently, the net magnetic moment of the sample increases and the macroscopic magnetism is enhanced. Following the introduction of Fe and Mn atoms into the lattice of Co${}_{66.6}$Si${}_{33.4}$, the orbit of Co is modified, and the hybridisation of the atomic orbital changes. After introducing the Fe and Mn dopants, the bond length changes, enhancing the magnetism. Figure 5 shows that the coercivity decreased after doping, and that of Co${}_{60.6}$Fe${}_{3}$Mn${}_{3}$Si${}_{33.4}$ reached a minimum value of 129.73 Oe.

These results show that the magnetic properties of Co${}_{66.6}$Si${}_{33.4}$ are enhanced after doping, and that the magnetic properties of Co${}_{60.6}$Fe${}_{3}$Mn${}_{3}$Si${}_{33.4}$ are excellent. The saturation magnetisation (Ms), coercivity (Hc), remanence magnetisation (Mr), and ratio of remanence to saturation magnetisation (Mr/Ms) of Co${}_{60.6}$Fe${}_{3}$Mn${}_{3}$Si${}_{33.4}$ are 36.3 emu/g, 129.73 Oe, 4.69 emu/g, and 0.16, respectively.

Figure 5a–d shows the Ms, Hc, Mr, and Mr/Ms of all samples. The x-axis represents the type of material, namely Co${}_{66.6}$Si${}_{33.4}$, Co${}_{60.6}$X${}_{6}$Si${}_{33.4}$, (X = Fe, Mn), or Co${}_{60.6}$Fe${}_{3}$Mn${}_{3}$Si${}_{33.4}$. The y-axis shows the Ms, Hc, Mr, and Mr/Ms values, respectively. In Figure 5a, the Ms values of Co${}_{66.6}$Si${}_{33.4}$, Co${}_{60.6}$X${}_{6}$Si${}_{33.4}$, (X = Fe, Mn), and Co${}_{60.6}$Fe${}_{3}$Mn${}_{3}$Si${}_{33.4}$ were 21.4, 27.9, 29.6, and 36.3 emu/g, respectively. Following doping, the magnetic saturation intensity improved, and that of Co${}_{60.6}$Fe${}_{3}$Mn${}_{3}$Si${}_{33.4}$ reaches the maximum value of 36.3 emu/g. When Co${}_{66.6}$Si${}_{33.4}$ is doped with Fe, the primary sources of magnetism are the d orbitals of Co and Fe; on the other hand, when Co${}_{66.6}$Si${}_{33.4}$ is doped with Mn, they are the d orbitals of Co and Mn.

The d orbital of Mn introduces a new magnetic source of magnetization atom, which enhances the coupling effect of the original system. When Co${}_{66.6}$Si${}_{33.4}$ is co-doped with Fe and Mn, the magnetic moment further increases, indicating that co-doping with Fe and Mn has a synergistic effect. Figure 5b compares the coercivities of Co${}_{66.6}$Si${}_{33.4}$, Co${}_{60.6}$X${}_{6}$Si${}_{33.4}$, (X = Fe, Mn), and Co${}_{60.6}$Fe${}_{3}$Mn${}_{3}$Si${}_{33.4}$ with that of Co${}_{66.6}$Si${}_{33.4}$, and indicates that the samples underwent varying degrees of deterioration.

The coercivities of Co${}_{66.6}$Si${}_{33.4}$ and Co${}_{60.6}$Fe${}_{3}$Mn${}_{3}$Si${}_{33.4}$ were 173.4, and 129.73 Oe, respectively. The Hc of Co${}_{60.6}$Fe${}_{3}$Mn${}_{3}$Si${}_{33.4}$ was the lowest among all samples. In Figure 5c, the Mr values of Co${}_{66.6}$Si${}_{33.4}$, Co${}_{60.6}$X${}_{6}$Si${}_{33.4}$, (X = Fe, Mn), and Co${}_{60.6}$Fe${}_{3}$Mn${}_{3}$Si${}_{33.4}$ exhibit slight differences. Further, the lower the Mr, the stronger the magnetic property. The Mr values of Co${}_{66.6}$Si${}_{33.4}$, Co${}_{60.6}$X${}_{6}$Si${}_{33.4}$, (X = Fe, Mn), and Co${}_{60.6}$Fe${}_{3}$Mn${}_{3}$Si${}_{33.4}$ were 7.8, 6.02, 4.67, and 4.69 emu/g, respectively.

As shown in Figure 5d, the Mr/Ms ratios of Co${}_{66.6}$Si${}_{33.4}$, Co${}_{60.6}$X${}_{6}$Si${}_{33.4}$, (X = Fe, Mn), and Co${}_{60.6}$Fe${}_{3}$Mn${}_{3}$Si${}_{33.4}$ were 0.38, 0.22, 0.16, and 0.13, respectively, exhibiting little in the way differences. In summary, the magnetic properties of Co${}_{60.6}$Fe${}_{3}$Mn${}_{3}$Si${}_{33.4}$ are significantly improved after doping; further, Co-doping had a significant effect on Co${}_{60.6}$Fe${}_{3}$Mn${}_{3}$Si${}_{33.4}$, whereby the saturation magnetisation reaches the maximum, its Hc was the lowest, and its Mr was remarkable.

**Figure 6:**
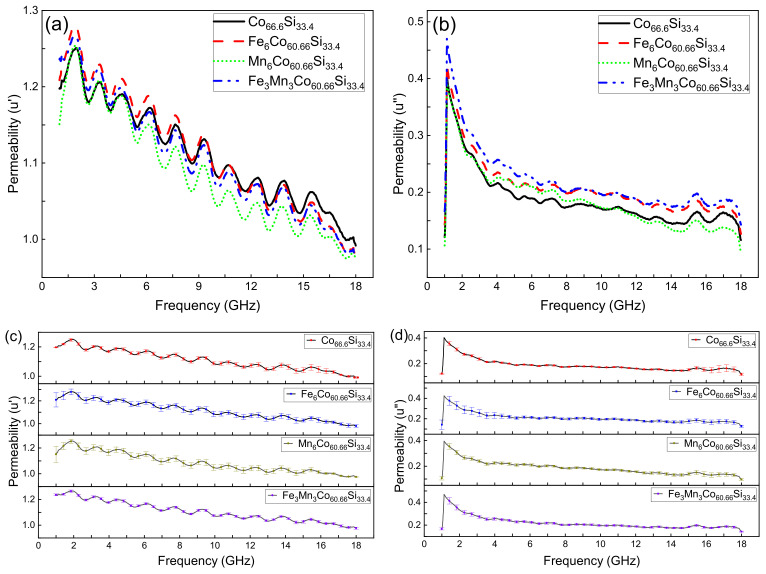
(**a**) Real permeability of Co${}_{66.6}$Si${}_{33.4}$, Co${}_{60.6}$X${}_{6}$Si${}_{33.4}$, (X = Fe, Mn), and Co${}_{60.6}$Fe${}_{3}$Mn${}_{3}$Si${}_{33.4}$; (**b**) Imaginary permeability of Co${}_{66.6}$Si${}_{33.4}$, Co${}_{60.6}$X${}_{6}$Si${}_{33.4}$, (X = Fe, Mn), and Co${}_{60.6}$Fe${}_{3}$Mn${}_{3}$Si${}_{33.4}$; (**c**) Real permeability of Co${}_{66.6}$Si${}_{33.4}$, Co${}_{60.6}$X${}_{6}$Si${}_{33.4}$, (X = Fe, Mn), and Co${}_{60.6}$Fe${}_{3}$Mn${}_{3}$Si${}_{33.4}$ (mean ± SD); (**d**) Imaginary permeability of Co${}_{66.6}$Si${}_{33.4}$, Co${}_{60.6}$X${}_{6}$Si${}_{33.4}$, (X = Fe, Mn), and Co${}_{60.6}$Fe${}_{3}$Mn${}_{3}$Si${}_{33.4}$ (mean ± SD).

Figure 6a,b, respectively, show the real (u’) and imaginary permeabilities (u”) of Co${}_{66.6}$Si${}_{33.4}$, Co${}_{60.6}$X${}_{6}$Si${}_{33.4}$, (X = Fe, Mn), and Co${}_{60.6}$Fe${}_{3}$Mn${}_{3}$Si${}_{33.4}$. Figure 6c,d, respectively, show the error bar of the real(u’) and imaginary permeabilities (u”) of Co${}_{66.6}$Si${}_{33.4}$, Co${}_{60.6}$X${}_{6}$Si${}_{33.4}$, (X = Fe, Mn), and Co${}_{60.6}$Fe${}_{3}$Mn${}_{3}$Si${}_{33.4}$ (mean ± SD). The real permeability represents the storage capacity of magnetic energy, while the imaginary permeability reflects the ability to lose magnetic energy [28]. As shown in Figure 6a,b, we chose Co${}_{66.6}$Si${}_{33.4}$ as an example because they all had the same trend. The u’ of Co${}_{66.6}$Si${}_{33.4}$ improved at 0.9–1.9 GHz, decreased at 1.9–2.68 GHz,, and reached a maximum at the 1.9 GHz.

The u’ of Co${}_{66.6}$Si${}_{33.4}$ fell and rose alternately, and there are ten peaks from 1.9 to 18 GHz. This figure shows a downward trend as a whole in the 1.9–18 GHz range. At 18–20 GHz, the permeability dropped to zero. The u’ of Co${}_{66.6}$Si${}_{33.4}$ started to appear at 1 GHz and reached its maximum immediately. Then it began to fall and reached 0 at 18 GHz.

As shown in Figure 6a,b, with increasing frequency, the u’ and u” of Co${}_{66.6}$Si${}_{33.4}$, Co${}_{60.6}$X${}_{6}$Si${}_{33.4}$, (X = Fe, Mn), and Co${}_{60.6}$Fe${}_{3}$Mn${}_{3}$Si${}_{33.4}$ gradually decreased, indicating that the storage capacity of magnetic energy and the ability to lose magnetic energy decrease with increasing frequency.

At low frequencies, the u’ and u” of Co${}_{66.6}$Si${}_{33.4}$, Co${}_{60.6}$Fe${}_{3}$Mn${}_{3}$Si${}_{33.4}$ increase, whereas at high frequencies, after doping, they changed only slightly. This phenomenon indicates that doping has a greater effect on the properties in the low-frequency region than in the high-frequency region. As shown in Figure 6c,d, the maximum standard deviation of the u’ of Co${}_{66.6}$Si${}_{33.4}$, Co${}_{60.6}$X${}_{6}$Si${}_{33.4}$, (X = Fe, Mn), and Co${}_{60.6}$Fe${}_{3}$Mn${}_{3}$Si${}_{33.4}$ is, respectively, 0.03264, 0.06129, 0.0615, 0.0091.

The maximum standard deviation of the u” of Co${}_{66.6}$Si${}_{33.4}$, Co${}_{60.6}$X${}_{6}$Si${}_{33.4}$, (X = Fe, Mn), and Co${}_{60.6}$Fe${}_{3}$Mn${}_{3}$Si${}_{33.4}$ is, respectively, 0.03264, 0.06129, 0.0615, 0.0091. This is because we measured three times, and in the process of each measurement, we made a new loop, thus, resulting in a certain error.

## 4. Conclusions

In this study, the effects of different dopants on the crystal structure, electrical conductivity, and magnetic properties of Co${}_{2}$Si were studied.

(1) Based on the PDF card (98-005-2281), a single phase of Co${}_{66.6}$Si${}_{33.4}$ was obtained by ball milling and sintering. The doped Co${}_{66.6}$Si${}_{33.4}$ did not form other phases, except for a phase of Co${}_{66.6}$Si${}_{33.4}$. There were slight differences between the measured and calculated grain sizes of Co${}_{66.6}$Si${}_{33.4}$, Co${}_{66.6}$Si${}_{33.4}$, Co${}_{60.6}$X${}_{6}$Si${}_{33.4}$, (X = Fe, Mn), and Co${}_{60.6}$Fe${}_{3}$Mn${}_{3}$Si${}_{33.4}$ (measured: 23, 21, 38, and 32 nm, respectively; calculated: 36.8, 34.4, 46.8, and 45.8 nm, respectively). This is due to the different methods employed (the data of XRD for calculation for calculation and TEM for measurements). Another reason is that magnetic materials affect the accuracy of the measured grain sizes.

(2) Under pressures of 1, 2, and 3 MPa, the resistivity of each samples was measured by a resistance meter. The conductivity was calculated by taking the reciprocals of resistivities. The effect of Fe doping on the conductivity was insignificant. The conductivities of Mn${}_{6}$Co${}_{60.6}$Si${}_{33.44}$ and Fe${}_{3}$Mn${}_{3}$Co${}_{60.6}$Si${}_{33.4}$ were superior to that of Co${}_{66.6}$Si${}_{33.4}$, indicating that the doping of Mn atoms has an influence on the electronic structure. This is because the entry of Mn atoms enhanced the electron mobility.

(3) The Co${}_{66.6}$Si${}_{33.4}$ alloy exhibited typical magnetic properties, which were improved after doping. At the same temperature, Ms increased, whereas Hc, Mr, and Mr/Ms decreased. This indicates that the magnetic properties of the Co${}_{66.6}$Si${}_{33.4}$ alloy are ameliorated after doping. Among the doping strategies employed, the effect of the co-doping of Fe and Mn was excellent, the resulting values of Ms, Hc, Mr, and Mr/Ms were 36.3 emu/g, 129.73 Oe, 4.69 emu/g, and 0.16, respectively.

(4) The storage capacity of magnetic energy and the ability to lose magnetic energy declined with increasing frequency for Co${}_{66.6}$Si${}_{33.4}$ and doped Co${}_{66.6}$Si${}_{33.4}$. This is because impedance matching also plays a role. If the impedance does not match, lesser electromagnetic waves will enter the material, resulting in no improvement in the storage capacity despite a decrease in the loss of magnetic energy. Doping affects the permeability in the low-frequency region more than in the high-frequency region.

(5) This work suggests alternative candidates for future magnetic functional materials. The low coercivities of Mn${}_{6}$Co${}_{60.6}$Si${}_{33.44}$ and Fe${}_{3}$Mn${}_{3}$Co${}_{60.6}$Si${}_{33.4}$ render them promising for use as audio and video magnetic head materials. The remanence characteristics of doped Co${}_{66.6}$Si${}_{33.4}$ may render it suitable for use in electronic transformers.

## Figures and Tables

**Table 1:** Lattice parameters of as-prepared Co${}_{66.6}$Si${}_{33.4}$, Co${}_{60.6}$X${}_{6}$Si${}_{33.4}$, (X = Fe, Mn), and Co${}_{60.6}$Fe${}_{3}$Mn${}_{3}$Si${}_{33.4}$.

	2θ°	B	d (Å)	hkl	a (Å)	b (Å)	c (Å)
	43.899	0.242	2.0545	021	0.70813	0.49439	0.37311
Co${}_{66.6}$Si${}_{33.4}$	45.859	0.028	1.9999	301	0.70813	0.48499	0.37734
	45.277	0.115	1.9771	121	0.78013	0.49361	0.37394
	43.902	0.239	2.0606	021	0.71019	0.49337	0.37480
Fe${}_{6}$Co${}_{60.6}$Si${}_{33.4}$	45.308	0.003	1.9999	301	0.71019	0.49582	0.37480
	45.874	0.100	1.9765	121	0.71019	0.49210	0.37536
	44.002	0.139	2.0562	021	0.71639	0.49615	0.36752
Mn${}_{6}$Co${}_{66.6}$Si${}_{33.4}$	45.248	0.057	2.0024	301	0.71639	0.49615	0.36752
	45.877	0.097	1.9764	121	0.71639	0.49615	0.36752
	44.107	0.034	2.0510	021	0.71273	0.49225	0.37100
Fe${}_{3}$Mn${}_{3}$Co${}_{60.6}$Si${}_{33.4}$	45.413	0.108	2.0252	301	0.71273	0.49225	0.37100
	45.865	0.109	2.0007	121	0.71273	0.49225	0.37100

**Table 2:** Average grain size by testing of as-prepared Co${}_{66.6}$Si${}_{33.4}$, Co${}_{60.6}$X${}_{6}$Si${}_{33.4}$, (X = Fe, Mn), and Co${}_{60.6}$Fe${}_{3}$Mn${}_{3}$Si${}_{33.4}$.

Structure	Co${}_{66.6}$Si${}_{33.4}$	Fe${}_{6}$Co${}_{60.6}$Si${}_{33.4}$	Mn${}_{6}$Co${}_{66.6}$Si${}_{33.4}$	Fe${}_{3}$Mn${}_{3}$Co${}_{60.6}$Si${}_{33.4}$
Size (nm)	23	21	38	32

**Table 3:** Conductivities of as-prepared Co${}_{66.6}$Si${}_{33.4}$, Co${}_{60.6}$X${}_{6}$Si${}_{33.4}$, (X = Fe, Mn), and Co${}_{60.6}$Fe${}_{3}$Mn${}_{3}$Si${}_{33.4}$.

Structure	Co${}_{66.6}$Si${}_{33.4}$	Fe${}_{6}$Co${}_{60.6}$Si${}_{33.4}$	Mn${}_{6}$Co${}_{66.6}$Si${}_{33.4}$	Fe${}_{3}$Mn${}_{3}$Co${}_{60.6}$Si${}_{33.4}$
1 MPa	280 (S/m)	297 (S/m)	30,002 (S/m)	17,500 (S/m)
2 MPa	512 (S/m)	649 (S/m)	68,900 (S/m)	24,300 (S/m)
3 MPa	183 (S/m)	190 (S/m)	111,000 (S/m)	32,000 (S/m)

## Data Availability

The data presented in this study are available on request from the corresponding author.
